# Beyond the Mainstream: Exploring Parent Protective Behaviors in Asian Families Experiencing Pediatric Chronic Pain

**DOI:** 10.3390/children12060742

**Published:** 2025-06-07

**Authors:** Yoonhee Kristina Kim, Ryan S. Ma, Rashmi P. Bhandari

**Affiliations:** Department of Anesthesiology, Perioperative, and Pain Medicine, School of Medicine, Stanford University, Stanford, CA 94305, USA; ryanma@stanford.edu (R.S.M.); rbhandar@stanford.edu (R.P.B.)

**Keywords:** pediatric pain, Asian American health, Asian American youth, parent protective behaviors, pain-related distress

## Abstract

Background/Objectives: Despite the striking prevalence of pediatric chronic pain (20% of youth), its impact on culturally diverse populations, particularly Asian families, remains underexplored. The existing literature on parent protective behaviors predominantly focuses on Non-Hispanic White (NHW) families, where such behaviors often exacerbate pain outcomes, therefore informing a core treatment target in clinical practice. This study aims to explore the role of parent protective behaviors in relation to global and pain-related distress in Asian families in comparison to NHW counterparts. Methods: A sample of 1415 youth (Asian: n = 236; NHW: n = 1179) aged 8 to 17 completed a survey prior to their evaluation at a tertiary pain clinic. Bivariate correlations and independent-sample *t*-tests were conducted to assess differences in anxiety, depression, pain-related distress, and parent protective behaviors between groups. Multiple regression analyses were used to determine whether parent protective behaviors moderated the relationship between global distress and pain-related outcomes. Results: Asian youth reported significantly lower pain intensity and pain interference than NHW youth, while Asian parents reported significantly higher protective behaviors. Parent protective behaviors moderated the association between global distress (depression and anxiety) and pain catastrophizing for Asian families only. A three-way interaction (ethnicity x parent protective behaviors, global distress, B = −0.22, *p* < 0.001; B = −0.18, *p* < 0.01) revealed that protective behaviors influenced the distress–pain catastrophizing link differently by ethnicity. Conclusions: Differences were observed in the Asian youth’s experience of pain in comparison to their NHW counterparts. This study highlights the importance of considering culturally nuanced approaches in treating pediatric chronic pain, particularly when working with Asian families.

## 1. Introduction

Pediatric chronic pain is a biopsychosocial phenomenon that is increasingly being recognized as a family matter [1]. With pediatric chronic pain affecting 20.8% of youth [2], the impact on family systems and communities is significant. Paired with the impact on family systems are the cultural and ethnic considerations regarding the experience of chronic pain. According to the Pew Research Center [3], Asian Americans are the fastest-growing racial group in the US. Therefore, it is important to understand the intersection of this vastly growing population and its representation—or lack thereof—in the pediatric chronic pain literature. We recognize the Asian/Asian American diaspora as highly diverse, encompassing many cultures. For this paper, we use the term “Asian” to broadly refer to individuals of Asian descent in the US, including those from various immigrant backgrounds.

Although the population of Asians in the US continues to grow, Asian families are consistently underrepresented in pain clinics and show low rates of help-seeking behaviors [4]. This trend is inconsistent with research demonstrating that Asians report lower levels of pain tolerance and higher ratings of pain unpleasantness [5] compared to their Non-Hispanic White (NHW) counterparts. Sensitivity to acute pain is a known risk factor for developing chronic pain [6], suggesting a potential gap in our understanding of chronic pain experiences among Asian youth and the low representation in pain clinics. Literature addressing the intersection of Asian families in pediatric chronic pain contexts is sparse. Limited research has highlighted Asians experiencing higher baseline and stress heart rate levels along with higher parental expectations and greater parental criticism in comparison to their NHW counterparts [5]. Consistently, Wang et al. [7] found that Asian Americans experience greater internalizing symptoms, family conflict, and lower self-esteem compared to their NHW peers. These symptoms are noteworthy as internalizing symptoms have been found to increase the likelihood of chronic pain in pediatric populations [8].

Parent protective behaviors, such as providing more pain-related attention to the child or allowing them to miss school, have been associated with greater functional disability [9], increased somatic symptoms [10], slower recovery from surgery [11], and more. Further, parent protective behaviors have demonstrated negative associations with youth overall function, school, pain intensity, and depression [12,13]. However, a major limitation of these studies is their limited generalizability, as most youth seeking pain treatment have been Caucasian or NHW [14]. Parent protective behaviors are shaped by family socialization practices, whereby in Asian families, parents have been found to often limit praise, favor criticism [7], and express warmth in more nonverbal and indirect ways [7,15]. The collectivistic cultural emphasis on stoicism and the suppression of personal expression [16] may contribute to increased physiological stress [17]. Given the unique dynamics within Asian families—characterized by both higher family conflict and stronger family cohesion—parent protective behaviors may have a distinct impact on the chronic pain experience of Asian youth. Possibly, explicit protective behaviors, which may provide validation and support, could help mitigate the emotional burden of chronic pain for Asian youth, counteracting the effects of stoicism and limited emotional expression. This study explores this psychosocial phenomenon by examining cultural influences on parenting. Since cultural beliefs shape parenting attitudes and behaviors [16], we will specifically focus on parent protective behaviors in the context of youth with chronic pain.

### Aims and Hypotheses

Our overarching aim is to explore how parent protective behaviors impact Asian youth in their experience of chronic pain. Specifically, the first aim is to compare reports of parent protective behaviors, youth global distress (anxiety and depression), and pain-related distress (pain intensity, pain catastrophizing, and pain interference) between Asian youth and their NHW counterparts. We hypothesized that Asians would report higher parent protective behaviors, greater global distress, and lower pain-related distress in comparison to NHW youth.

The second aim is to investigate how parent protective factors impact the relationship between youth global distress and pain-related distress in Asian versus NHW families. We hypothesized that the impact of protective factors would differ by ethnicity: in NHW families, parent protective behaviors are expected to moderate, and thus strengthen, the relationship between youth global distress and pain-related distress, consistent with the existing literature [9], while in Asian families, we predict that greater parent protective behaviors will buffer the impact that global distress has on pain-related distress.

## 2. Materials and Methods

### 2.1. Participants

Data were retrospectively extracted from the Pediatric-Collaborative Health Outcomes Information Registry (Peds-CHOIR). The pediatric adaptation of the Collaborative Health Outcomes Information Registry (Peds-CHOIR) is a free, open-source, flexible learning health care system (LHS) that meets the call by the Institute of Medicine for the development of national registries to guide research and precision pain medicine. Using a web-based interface, the registry has capabilities to capture data at each clinic visit; display graphical, real-time results that inform point-of-care decisions; and track patient treatment responses longitudinally [18]. The registry is a part of the clinical infrastructure and is completed by all patients who are seen in the tertiary Pediatric Pain Management Clinic (PPMC) at Stanford Children’s Health, California, an interdisciplinary clinic. Data included in this study were cross-sectional and completed before the initial evaluation at the PPMC. The data included in this analysis were extracted from June 2014 to August 2023 and included patients aged 8–17 years old. Furthermore, patients included in this study self-identified as Non-Hispanic White (NHW; *n* = 1389) or Non-Hispanic Asian (*n* = 254). The Peds-CHOIR dataset at large was used for previous studies [19,20,21], but none to date have specifically explored the comparison between Non-Hispanic White (NHW) vs. Non-Hispanic Asian youth.

### 2.2. Measures

#### 2.2.1. Demographic Questionnaire

The Demographics and Pain History Questionnaire includes patient demographic information including age, sex, gender identity, race, and ethnicity.

#### 2.2.2. Average Pain Intensity

An 11-point numeric rating scale from 0 (no pain) to 10 (worst pain possible) is used to quantify pain intensity (REF) and has demonstrated validity and reliability for self-report of pain intensity in children and adolescents [22].

#### 2.2.3. PROMIS Pediatric Patient and Caregiver (Proxy) Outcome Measures

PROMIS Pain Interference, Anxiety, and Depression measures were used in this study. The PPMC uses the PROMIS computer adaptive testing (CAT) version and item response theory (IRT) to understand patient reports of mental, physical, and social functioning. CAT administers items from an item bank based on responses to earlier items, which reduces the number of items administered to patients but has been shown to improve precision. The final score, however, has been shown to reflect the construct being evaluated. This measure was validated in a representative sample of US patients with chronic conditions and their families, as well as in the general population through schools and primary care clinics.

PROMIS scores are centered at a mean of 50 and a standard deviation of 10, using a T-score distribution. The pediatric PROMIS measures assess physical, social, and psychological functioning domains in children 8 to 17 years old. Responses are on a 1–5 scale (1 = “Never/Not able to do” and 5 = “Almost always/With no trouble”). Higher scores indicate more of the construct being measured.

#### 2.2.4. Pain Interference

The PROMIS pediatric pain assessment asks questions to understand the impact of pain on social, psychological, and physical functioning (e.g., “It was hard for me to have fun when I had pain”) over the past 7 days. Higher scores indicate increased pain interference. 

#### 2.2.5. Pediatric Anxiety

The PROMIS pediatric anxiety questionnaire assesses patient worries, fears, and nervousness (e.g., “In the past 7 days I felt worried”). Elevated scores indicate higher levels of anxiety.

#### 2.2.6. Pediatric Depression

The PROMIS pediatric depression questionnaire assesses patients’ negative mood, social cognition, and self-perceptions (e.g., “In the past 7 days I felt alone”). Elevated scores indicate more depressive symptoms.

#### 2.2.7. Parental Protective Behavior

The Adult Responses to Children’s Symptoms (ARCS) Questionnaire is a 13-item assessment that seeks to understand parent/guardian responses to children’s symptoms and behaviors. Higher scores indicate more protective behaviors and items are scored on a scale from 0 = “Never” to 4 = “Always”. Examples include, “When your child is in pain, how often do you... spend more time than usual with your child, stay home from work or come home early (or stay home instead of going out or running errands), and let your child sleep later than usual in the morning?” There are no clinical ranges for parent protective behaviors; higher scores denote greater parent protective behaviors.

#### 2.2.8. Pain Catastrophizing Scale (PCS-C)

Patient pain catastrophizing for children (PCS-C) was analyzed, which seeks to understand child and adolescent feelings of helplessness and catastrophic thoughts. Examples of questions asked are “When I am in pain, it’s awful and I feel that it overwhelms me”, “When I am in pain, I want the pain to go away,” and “When I am in pain, I am afraid that the pain will get worse”. The PCS-C questionnaire is a 13-item measure rated with 0 = “Not at all true” to 4 = “Extremely true”. Higher scores indicate heightened pain catastrophizing with clinical bands as follows: low = 0–14, moderate = 15–25, and high ≥ 26.

#### 2.2.9. Global vs. Pain-Related Distress

Global distress was separately measured by the Pediatric Anxiety and Pediatric Depression scales. Similarly, pain-related distress also comprised three distinct measures: Average Pain Intensity, Pain Interference, and Pain Catastrophizing scales. These scales were not collapsed into one value (i.e., “global distress value” or “pain-related distress value”) and were analyzed separately.

## 3. Data Analyses

Data were analyzed using SPSS 29.0.2.0. Prior to conducting the primary analyses, the frequency and pattern of missing values were assessed. Missing values were deleted from the analyses. For the NHW population (*n* = 1389), between 55 and 210 cases were excluded across variables. For the Asian population (*n* = 254), between 10 and 18 cases were excluded per variable due to missing values. To assess the pattern of missing data, Little’s MCAR (Missing Completely at Random) test was conducted across variables for each group, including pain intensity, pain interference, anxiety, depression, pain catastrophizing, and parent protective behaviors. The test yielded a significant chi-square value for the Asian population (χ^2^(16) = 26.54, *p* = 0.047) and for the White population (χ^2^(36) = 96.32, *p* < 0.001), indicating non-MCAR data. Of note, missing data for the parent protective behaviors variable (13.9%) were not MCAR. When parent protective behaviors were excluded for the White population only, the data met MCAR assumptions. To ensure robustness and, to ensure that the results were not an artifact of the method chosen for handling missing data, a sensitivity analysis was conducted by comparing results from listwise deletion and mean imputation. The results revealed key findings to be consistent across both listwise deletion and series mean imputation, indicating that the results were not highly sensitive to the missing data. Consequently, listwise deletion was used to maintain the full variability of the data. All variables were then mean-centered to reduce multicollinearity.

Aim #1: Compare reports of parent protective behaviors, youth global distress (anxiety and depression), and pain-related distress (pain intensity, pain catastrophizing, and pain interference) between Asian youth and their NHW counterparts.

To address the first aim, an independent-sample t-test was conducted to compare Asian and Non-Hispanic White (NHW) youth in their reports of pain-related distress, global distress, and parent protective behaviors. Due to unequal sample sizes between Asian and NHW youth, Levene’s test for homogeneity of variances was conducted to assess whether the assumption of equal variances is met for each variable across the two groups.

Aim #2: Investigate how parent protective factors impact the relationship between youth global distress and pain-related distress in Asian versus NHW families.

For the second aim, six hierarchical multiple regression analyses were conducted for each ethnic group to understand the extent to which parent protective behaviors interacted with the relationship between global distress (anxiety or depression) and pain-related distress (pain intensity, pain catastrophizing, or pain interference). Each model examined one combination of global distress (either anxiety or depression) and parent protective behaviors as independent variables, with a pain-related distress variable (i.e., pain intensity, pain interference, or pain catastrophizing) as the dependent variable. Parent protective behaviors were tested as the moderator (interaction with global distress) across all models. For each model, the following variables were entered for the stepwise linear regression analysis: In the first step, demographic variables associated with the independent and dependent variables—sex and patient age—were added as control variables. In the second step, the independent variables of anxiety or depression and parent protective behaviors were added. In the third step, the interaction term between parent protective behaviors and global distress was included to assess the moderating effect.

Finally, for data containing both ethnic groups, a post hoc model with a 3-way interaction was conducted only for the significant moderation model. Thus, the 3-way interaction was fitted to investigate the relationship between ethnic group, parent protective behaviors, and global distress on pain catastrophizing. This model also contained anxiety or depression, parent protective behaviors, and their two-way interaction.

* *p* < 0.05 was considered statistically significant throughout.

## 4. Results

There were 1179 NHW patients and 236 Asian patients included in the total sample. Table 1 presents the bivariate correlations for all variables for Asian families and NHW families.

For Aim 1, Asian youth reported lower average pain intensity (*M* = 5.16, *SD* = 2.46) and pain interference (*M* = 57.63, *SD* = 8.16) compared to NHW youth, as indicated in Table 2. Cohen’s d effect sizes indicated large, statistically significant differences (*t*(1177) = −2.97, *p* < 0.01; and *t*(1177) =−3.46, *p* < 0.001, respectively). Asian parents reported significantly higher parent protective behaviors compared to NHW parents (*M* = 1.79, *SD* = 0.95), with moderate and significant effect sizes (*t*(1177) = 4.53, *p* < 0.001). Levene’s test indicated that the assumption of homogeneity of variances was met for all dependent variables (*p* > 0.05), except for parent protective behaviors (F = 11.499, *p* < 0.001). The unequal variance assumption was violated for parent protective behaviors, and therefore Welch’s correction was applied. Using Welch’s *t*-test to account for this, Asians exhibited significantly higher parent protective behaviors than Whites (*t*(300.595) = 4.492, *p* < 0.001, mean difference = 0.296, 95% CI [0.166, 0.426]). There were no significant differences between Asian and NHW youth in pain catastrophizing, anxiety, and depression.

For Aim 2, none of the moderation effects of all six models for the NHW population were statistically significant. Two of the moderation effects for the Asian population were statistically significant, as indicated in Table 3. Parent protective behaviors moderated the relationship between anxiety and pain catastrophizing (B = 0.19, SE = 0.06, β = 0.18, *p* = 0.002). Secondly, parent protective behaviors moderated the relationship between depression and pain catastrophizing (B = 0.13, SE = 0.06, β = 0.12, *p* = 0.038).

**Figure 1:**
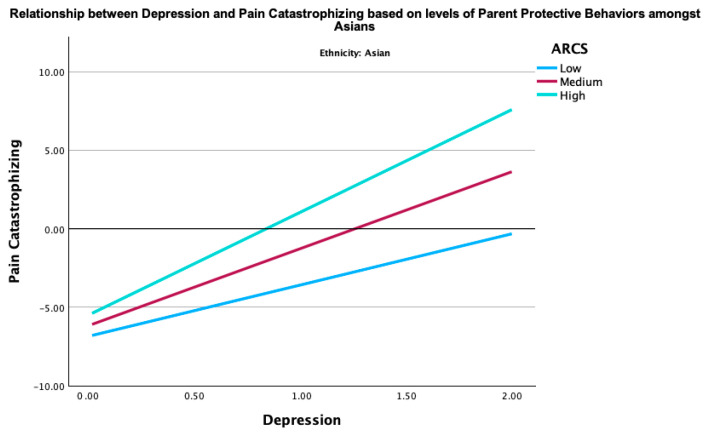
Interaction of depression in predicting youth pain catastrophizing by levels of parent protective behaviors for Asian youth. ARCS = parent protective behaviors.

**Figure 2:**
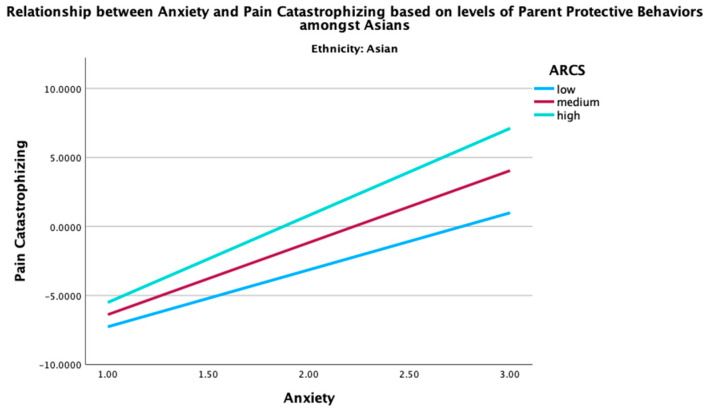
Interaction of anxiety in predicting youth pain catastrophizing by levels of parent protective behaviors for Asian youth. ARCS = parent protective behaviors.

**Figure 3:**
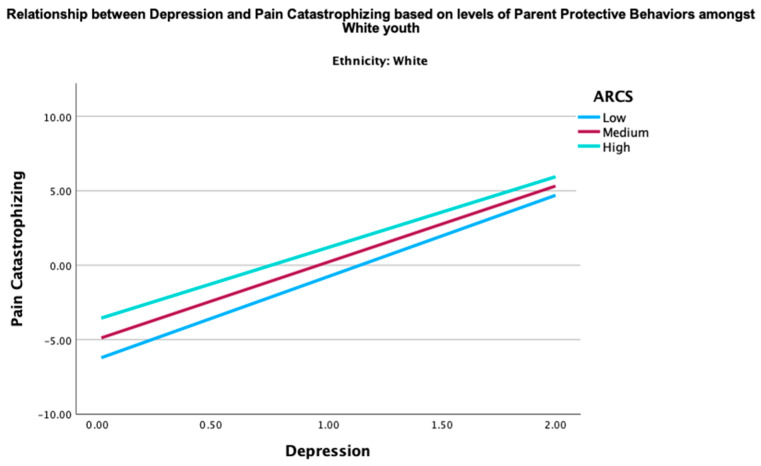
Interaction of depression in predicting youth pain catastrophizing by levels of parent protective behaviors for NHW youth. ARCS = parent protective behaviors.

**Figure 4:**
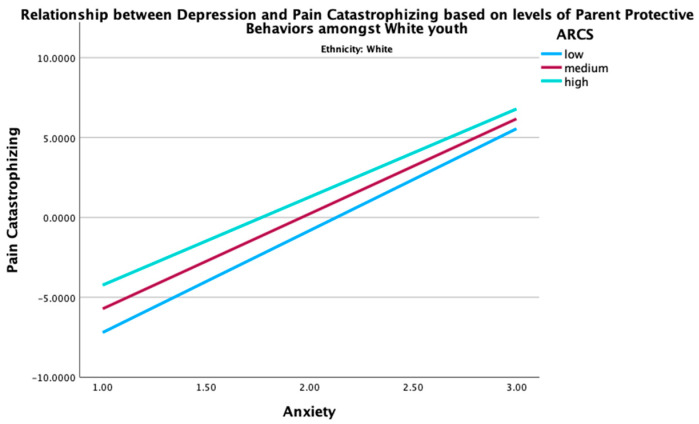
Interaction of anxiety in predicting youth pain catastrophizing by levels of parent protective behaviors for NHW youth. ARCS = parent protective behaviors.

Given the significant moderation effect of the parent protective behaviors in the relationship between global distress and pain catastrophizing in Asian youth, two post hoc three-way interaction analyses were conducted to examine how ethnicity, parent protective behaviors, and global distress (depression or anxiety) together influence pain catastrophizing. The three-way interactions between ethnicity, parent protective behaviors, and global distress (depression (B = −0.22, *p* < 0.001) and anxiety (B = −0.18, *p* = 0.008) were both significant. These findings indicate that the combined effect of parent protective behaviors and global distress on pain catastrophizing was moderated by ethnicity. Specifically, the interaction term between ethnicity and parent protective behaviors revealed that the association between parent protective behaviors and pain catastrophizing was stronger among Asians, as elucidated in Figure 1 and Figure 2, in comparison to their NHW counterparts (Figure 3 and Figure 4). This may imply that Asian youth with higher levels of anxiety or depression are more sensitive to the effects of parent protective behaviors on pain catastrophizing than their NHW counterparts.

Figure 1, Figure 2, Figure 3 and Figure 4 show the post hoc 3-way analysis of ethnicity, parent protective behaviors, and global distress on pain catastrophizing.

Figure 1 and Figure 3 represent the Asian demographic, while Figure 2 and Figure 4 represent the NHW demographic. Levels of parent protective behaviors were plotted by selecting values 1 SD above (high parent protective behaviors), −1 to 1 SD (medium parent protective behaviors), and 1 SD below (low parent protective behaviors).

## 5. Discussion

The current study provides important insights into the experience of pediatric chronic pain within Asian families, a growing demographic that has been underrepresented in previous research. One of the most striking findings in the current study was the significant ethnic differences in pain intensity, pain interference, and parent protective behaviors. Asian youth reported lower levels of pain intensity and pain interference compared to their NHW counterparts, with the differences being large and statistically significant. This suggests that cultural factors may play a role in how pain is perceived and reported, aligning with their low representation in tertiary pain clinics. Further, Asian parents reported significantly higher levels of parent protective behaviors than NHW parents. These behaviors likely include actions such as shielding children from stressful situations, providing emotional support during pain episodes, and possibly limiting exposure to pain-inducing activities. The finding that Asian parents engage in more protective behaviors is consistent with cultural values that emphasize family cohesion [23] and parental involvement [16], which may be more pronounced in some Asian cultures compared to Western ones. This could reflect cultural norms around parenting, where greater parental control or care is perceived as a form of protection or support for children [16], particularly when managing distress or pain. However, pain catastrophizing, depression, and anxiety were not significantly different between the two racial/ethnic groups.

Secondly, for Asians only, parent protective behaviors moderated the relationship between global distress and pain catastrophizing. In contrast to our hypothesis—that greater parent protective behaviors might buffer or mitigate the effects of global distress on pain-related outcomes—our results indicate that for Asian youth, the association between global distress and pain catastrophizing is stronger when parent protective behaviors are high. Furthermore, the significant three-way interaction among ethnicity, parent protective behaviors, and global distress suggests that the influence of parent protective behaviors on pain catastrophizing is not uniform across ethnic groups; it is notably stronger among Asian youth. These findings suggest cultural differences in the interplay between parenting practices, psychological distress, and pain-related outcomes. This may be reflective of the “Striving, Persistent, Behavioral Style” (SPBS) [17], which posits that while Asian adolescents are driven and high achieving, they also tend to suppress emotional expression due to cultural values such as stoicism [18]. This aligns with existing literature showing that Asian youth are more prone to internalizing symptoms, such as anxiety and depression, than their NHW peers [7,24].

When considered alongside existing research on Asian youth and mental health, these findings reveal a narrative that diverges from patterns typically observed in NHW youth. Although Asian youth report higher rates of mental health challenges, they also report lower pain intensity and prevalence, accompanied by greater levels of parent protective behaviors. This divergence highlights the importance of considering cultural frameworks and coping mechanisms, such as stoicism and collectivistic values, that may influence healthcare-seeking behaviors and pain reporting.

Several explanations may help frame the results of our study. Broadly, Western analysis characterizes Asian families as differing from Caucasian families due to a collectivistic mindset (as opposed to individualistic), in which Asians prefer interdependence and group harmony over independence and meeting individual needs [7]. This collectivistic framework may partly explain lower rates of formal help-seeking (e.g., from healthcare providers) compared to other ethnic groups [25] often due to a sense of familial obligation [16]. Rooted in the Confucian value of familism, which positions the family as society’s foundation [26], this cultural value may discourage help-seeking when it appears to prioritize individual needs over the needs and concerns of the family. Additionally, help-seeking behaviors amongst Asians may be influenced by preferences for complementary and alternative medicine (CAM) treatment [27,28], a lack of knowledge of healthcare access [4], perception of pain as a natural part of life rather than a treatable condition [29,30], or the adoption of stoicism [30]

Additionally, as a society, we may be incorrectly assessing the chronic pain experience among Asians due to the influence of the model minority myth, both systemically and individually internalized. The systemically internalized model minority myth has made a faulty assumption that there is a reduced urgency to address the distress experienced by Asians, due to their high functionality, resilience, and academic competence. This assumption is reinforced by low levels of pain prevalence, pain intensity, and pain interference. A personally internalized model minority myth may also shape how Asian youth perceive, cope with, and report pain, leading them to minimize their experiences in favor of group needs, consistent with a collectivistic mindset [16,24]. Together, these systemic and individual expressions of the model minority myth are cyclical; research on Asian populations in the health literature is sparse due to the perception that there is not a need for such research, survey measures administered in clinical spaces have not sought to ask questions specific to the Asian experience and different expressions of pain, and Asians may align their externalizing expressions of pain with societal and personal expectations. Stoicism, reinforced by the model minority myth, may further contribute to this phenomenon, as evidenced by a few striking pediatric studies. For example, Thai children relied significantly more on covert coping methods (e.g., distraction via thinking of favorite things), whereas American children reported more explicit coping methods (e.g., screaming) [31,32]. Similarly, when undergoing immunization, Japanese babies were significantly more likely to demonstrate low behavioral but high cortisol responses compared to White infants who were more likely to display high behavioral but low cortisol responses [32,33]. These studies align with SPSB’s [17] conceptualization of emotion suppression, prevention motivation, and unmodulated persistence. Furthermore, Asians who function in the SPBS framework are nested under contexts such as oppression, structural racism, and chronic stress, which increase allostatic load and risk for health problems such as pediatric chronic pain.

However, it is also important to note resiliency factors that may be unique to Asian families. Asian families may pull from resiliency factors such as greater openness to complementary/integrative management procedures [28,34], reliance on community/family systems [35], and overall self-management protocols to navigate pain [36]. The SPBS also denotes grit, perseverance, and resilience as an undeniable component of the framework [17], which, when nested in more supportive systems, may highly serve the Asian demographic. These findings underscore the importance of cultural context when considering interventions for youth with chronic pain and distress. For Asian youth, interventions that target family dynamics—particularly those aimed at optimizing parent-child interactions—may be critical. Tailored interventions might include training for parents to provide supportive but not overprotective behaviors, thereby mitigating the amplifying effect of high global distress on pain catastrophizing. Clinically, it may be particularly important to attend to Asian youth with higher reported distress, as the results indicate that they may be more negatively impacted by the interaction of parent protective behaviors, thus exacerbating pain catastrophizing. A personalized approach to treatment that considers cultural values, family dynamics, and individual coping strategies is essential in providing optimal care for youth facing both depression and chronic pain.

Several limitations must be acknowledged. First, the cross-sectional design precludes causal inferences. Future longitudinal studies are needed to examine how these relationships develop over time. Second, the disparity in group sizes, particularly the smaller Asian sample, warrants caution; future studies should aim to recruit larger and more geographically diverse Asian samples. Third, the study’s measures (e.g., the unidimensional assessment of parent protective behaviors) may not fully capture the culturally nuanced behaviors unique to different Asian subgroups. Protective behaviors may be influenced by culturally embedded values such as filial piety, collectivism, and the prioritization of academic success, which are not adequately represented in standard Western-developed measures. Without accounting for these contextual and developmental variations, the measure may inadvertently oversimplify or misrepresent behaviors to a single measure that is “protective behaviors”. Future research should consider multi-faceted ways to capture parental responses, intent, and behaviors in the form of culturally validated measures and qualitative interviews. Finally, the label “Asian” encompasses a highly heterogeneous group, tracing its roots to more than 20 different countries; future research should explore intra-Asian differences, including the roles of immigration status, language proficiency, and acculturation. Future studies may also examine differences in parent protective behaviors by age group of youth experiencing chronic pain to better understand the interplay of developmental milestones with ways that parenting practices influence pain outcomes.

Limitations notwithstanding, this study is the first to investigate pediatric chronic pain in a large sample of Asian youth, revealing nuanced interactions among cultural values, parent protective behaviors, and psychological and pain-related distress. Our findings challenge the assumption that Asian youth are at lower risk for pain-related issues simply because they report lower pain intensity and interference. In fact, our results suggest that youth experiencing greater levels of anxiety and depression may be more sensitive to parent protective behaviors, thus intensifying pain catastrophizing. These insights underscore the need for culturally informed approaches in both research and clinical practice, ensuring that interventions are sensitive to the unique experiences and challenges faced by Asian youth with chronic pain. Future directions should investigate the mechanism of parent protective behaviors in Asian families and the experience of chronic pain. Specifically, the severity of mental health may differentially impact the way in which parent protective behaviors are internalized and therefore have a different influence on pain-related outcomes. Further research is needed to gather experiences of chronic pain in the community to assess culturally informed healthcare barriers. Qualitative studies are essential to lean into the lived, rich, and heterogeneous experiences of Asians, identifying vulnerability and resiliency factors that influence access to tertiary pain clinics, and examining familial dynamics in pediatric chronic pain. A deeper understanding of these elements will inform more inclusive, culturally responsive pain management strategies, ultimately improving care for underserved populations.

## Figures and Tables

**Table 1:** Bivariate correlations for study variables of Asian and NHW samples.

	1.	2.	3.	4.	5.	6.
1. Pain Intensity	-	0.33 **	0.40 **	0.22 **	0.20 **	0.09
2. PCS ^a^	0.32 **	-	0.54 **	0.57 **	0.51 **	0.28 **
3. Pain Interference	0.42 **	0.50 **	-	0.50 **	0.46 **	0.28 **
4. Anxiety	0.22 **	0.57 **	0.42 **	-	0.79 **	0.20 **
5. Depression	0.18 **	0.51 **	0.43 **	0.72 **	-	0.15 *
6. Parent Protect ^b^	0.16 **	0.18 **	0.27 **	0.16 **	0.21 **	-

* *p* < 0.05; ** *p* < 0.01; ^a^ PCS = Pain Catastrophizing Scale; ^b^ Parent Protect = Parent Protective Behaviors; Note. Coefficients appearing above the diagonal correspond to the Asian sample (*n* = 236); coefficients below the diagonal correspond to the NHW sample (*n* = 1179).

**Table 2:** Means, standard deviations, and independent-sample t-test results for Aim 1.

	NHW		Asian		
	M	SD	M	SD	*t* Test
1. Pain Intensity	5.64	2.21	5.16	2.46	−2.97 **
2. PCS	27.74	10.87	26.78	11.66	−1.21
3. Pain Interference	59.67	8.14	57.63	8.16	−3.46 ***
4. Anxiety	53.89	10.46	52.82	10.61	−1.40
5. Depression	55.77	10.81	54.48	10.99	−1.65
6. Parent Protective	1.49	0.77	1.79	0.95	4.53 ***

** *p* < 0.01; *** *p* < 0.001.

**Table 3:** Multiple regression analyses examining the effect of global distress (anxiety and depression) and parent protective behavior on pain catastrophizing for Aim 2.

		Anxiety x Parent Protective Behaviors on Pain Catastrophizing				Depression x Parent Protective Behaviors on Pain Catastrophizing			
		B	*SE*	Beta	t-Value	B	*SE*	Beta	t-Value
1	Constant								
	Patient Age	0.24	0.32	0.05	0.76	0.24	0.32	0.05	0.76
	Gender	−2.60	1.66	−0.11	−1.57	−2.60	1.66	−0.11	−1.57
2	Constant								
	Patient Age	−0.04	0.28	−0.01	−0.16	−0.04	0.27	−0.01	−0.14
	Gender	−0.76	1.43	−0.03	−0.54	−0.32	1.40	−0.01	−0.23
	Distress	0.49	0.06	0.46	7.99 ***	0.55	0.06	0.50	8.80 ***
	Parent Protect ^a^	2.51	0.71	0.20	3.56 ***	2.17	0.69	0.18	3.12 **
3	Constant								
	Patient Age	−0.08	0.27	−0.02	−0.30	−0.04	0.27	−0.01	−0.15
	Gender	−0.89	1.40	−0.04	−0.63	−0.42	1.39	−0.02	−0.30
	Distress	0.45	0.06	0.42	7.28 ***	0.50	0.07	0.45	7.30 ***
	Parent Protect	2.81	0.70	0.23	4.04 ***	2.40	0.70	0.20	3.44 ***
	Moderation	0.19	0.06	0.18	3.21 **	0.13	0.06	0.12	2.07 *

* *p* < 0.05; ** *p* < 0.01; *** *p* < 0.001; ^a^ Parent protect = Parent protective behavior.

## Data Availability

The deidentified dataset is not publicly available due to the Stanford Medicine policy regarding patient privacy and data sharing. Requests to access the dataset should be directed to Dr. Kim (yoonheek@stanford.edu) to inquire regarding the possibility of a data-sharing agreement.
